# Supplementary material for: False head complexity and evidence of predator attacks in male and female hairstreak butterflies (Lepidoptera: Theclinae: Eumaeini) from Mexico
Source: PeerJ. 2019 Jun 25;7:e7143. doi: 10.7717/peerj.7143 (PMC6598652; doi:10.7717/peerj.7143)
Supplement: Appendix S2 [file peerj-07-7143-s002.docx]

**APPENDIX 2.** Akaike Information Criterion tables for the Generalized Linear Models.

Table 1. AIC values for generalized linear mixed models testing effect of false head complexity and scientific collection of origin on symmetrical damage at the species level. Models are shown in order of increasing AIC, and ∆AIC is with respect to the model with the lowest AIC value. FHC=False Head Components

| Explanatory variables in model | AIC | ∆AIC |
| --- | --- | --- |
| FHC + Collection | 466.9 | 0 |
| FHC | 469.4 | 2.5 |
| Collection | 477.9 | 11 |
| (null model) | 479.9 | 13 |

Table 2. AIC values for generalized linear mixed models testing effect of false head complexity and scientific collection of origin on symmetrical damage at the genus level. Models are shown in order of increasing AIC, and ∆AIC is with respect to the model with the lowest AIC value. FHCavg=False Head Components averaged by genus.

| Explanatory variables in model | AIC | ∆AIC |
| --- | --- | --- |
| FHC avg+ Collection | 265.0 | 0 |
| FHCavg | 271.0 | 6 |
| Collection | 271.6 | 6.6 |
| (null model) | 276.3 | 11.3 |

Table 3. AIC values for generalized linear mixed models testing effect of false head complexity (FHC), scientific collection of origin, sex, and the interaction of sex and FHC (FHC*Sex) on symmetrical damage at the species level. Models including all ten possible combinations of the explanatory variables were constructed and are shown here in order of increasing AIC. ∆AIC is with respect to the model with the lowest AIC value.

| Explanatory variables in model | AIC | ∆AIC |
| --- | --- | --- |
| Collection + FHC | 445.2442 | 0 |
| Collection + FHC + Sex +FHC*Sex | 445.9523 | 0.7081345 |
| FHC | 446.9211 | 1.6768849 |
| Collection + FHC + Sex | 447.1801 | 1.9358791 |
| FHC + Sex +FHC*Sex | 447.6836 | 2.4394246 |
| FHC + Sex | 448.8458 | 3.6016146 |
| Collection | 450.5486 | 5.3043929 |
| (null model) | 452.0342 | 6.7899925 |
| Collection + Sex | 452.4883 | 7.2441656 |
| Sex | 453.9619 | 8.7176971 |

Table 4. AIC values for generalized linear mixed models testing effect of false head complexity (averaged by genus, FHCavg), scientific collection of origin, sex, and the interaction of sex and FHCavg (FHCavg*Sex) on symmetrical damage at the genus level. Models including all ten possible combinations of the explanatory variables were constructed and are shown here in order of increasing AIC. ∆AIC is with respect to the model with the lowest AIC value.

| Explanatory variables in model | AIC | ∆AIC |
| --- | --- | --- |
| Collection + FHCavg | 311.6418 | 0 |
| Collection | 313.5239 | 1.882127 |
| Collection + FHCavg + Sex | 313.5957 | 1.953904 |
| FHCavg | 313.7577 | 2.115952 |
| Collection + FHCavg + Sex + FHCavg*Sex | 314.8386 | 3.196774 |
| (null model) | 315.2599 | 3.618151 |
| Collection + Sex | 315.4935 | 3.851707 |
| FHCavg + Sex | 315.7077 | 4.065952 |
| FHCavg + Sex +FHCavg*Sex | 316.9778 | 5.336019 |
| Sex | 317.2256 | 5.583854 |
